# Practical Hints

**Published:** 1909-08-14

**Authors:** 


### PRACTICAL HINTS.

### Leaking Water-cooling System.

An excellent method of repairing a leak in pipes or joints in the water-cooling system of a petrol-motor is to bind round the part leaking a length of string that has previously been soaked in oil. A very satisfactory temporary repair can be effected in this way. The string should first be soaked in oil—linseed for preference. Failing this, thick lubricating oil can be used, and the string should be wound round the joint with the coils kept as close together as possible. The start and finish of the coil should be some little distance on each side of the point at which the leak occurs. The winding should if possible consist of two or three layers of string. In the case of a leak in the tank, if the fracture is sufficiently large some tow can be made by picking a piece of string, soaking it in oil, and packing it into the joint by means of a chisel or a strong blade of a penknife. White lead is, of course, at all times preferable to oil, and if a piece of tape can be used in conjunction with this a satisfactory and permanent repair can be effected. Whilst writing about the cooling system I would like to draw attention to the fact that in cases where engines overheat without apparent cause, and where there is no doubt that the water is circulating in a perfect manner, it will be found that hard water has been used for cooling purposes. Well waters should never be used, as, almost without exception, they contain large quantities of mineral salts in solution. An invariable practice of using soft water only should be made if cooling troubles are to be avoided. Care should be taken, however, to strain soft water so as to prevent the admission of particles of foreign matter such as are usually found floating on the surface of rainwater.

### Driving Over Stones.

It is very generally known that driving over newly laid patches of stones can cause considerable damage to tyres. Hence the problem of how to pass over with the least possible amount of harm is of great moment to the novice. Many drivers drop down to bottom speed and go over slowly. This, although much less risky than driving over fast, frequently results in the tyres being badly cut. The best way is to let the car run at speed right up to the patch of loose metal and take the clutch out before the front wheels strike the stones, when sufficient momentum will be left to carry it over the average-sized patch of stones. By this method the wheels are relieved of all driving strains and the tyres are less likely to be damaged, since they simply roll over the stones and are not subjected to any other severe strains than the dead load. If, on account of the length of the patch of road metal or an adverse gradient the car cannot be got to roll over, nothing remains but to drop down to the low speed and drive over slowly.

### "Misfiring."

A most common cause of misfiring is a sparking-plug fouled with soot. Some drivers are frequently troubled in this way, but an external spark gap in the secondary circuit is said to overcome the difficulty. Prevention being better than cure, one should see that over-lubrication is not taking place.

"Viator."

